# Benefits and Risks Associated with Meat Consumption during Key Life Processes and in Relation to the Risk of Chronic Diseases

**DOI:** 10.3390/foods11142063

**Published:** 2022-07-12

**Authors:** Carlotta Giromini, D. Ian Givens

**Affiliations:** 1 Department of Veterinary and Animal Science, University of Milan, 20133 Milano, Italy; 2 Institute for Food, Nutrition and Health, University of Reading, Reading RG6 6EU, UK; d.i.givens@reading.ac.uk

**Keywords:** red meat, processed meat, anaemia, chronic diseases, dementia, bone health, pregnancy

## Abstract

Red meat has been an important part of the diet throughout human evolution. Overall, when included as part of a healthy and varied diet, red meat can provide a rich source of bioavailable essential nutrients and high biological value protein. The present paper discusses the dietary role/impact of red and processed meat, with some reference to the relative effect of white meat, in a range of chronic conditions including iron-deficiency anaemia, cardiovascular diseases (CVD), cancer and dementia. The role of red meat in relation to key physiological conditions such as maintaining skeletal muscle and bone health and during pregnancy is also discussed. The inclusion of lean red meat in a healthy, varied diet may be beneficial during these critical conditions. There is however increasing evidence that red meat and especially processed meat are associated with increased risks of CVD, cancer and dementia whereas white meat is neutral or associated with a lower risk. There now seems little doubt that processed and unprocessed meat should have separate public dietary guidance.

## 1. Introduction

**Figure 1:**
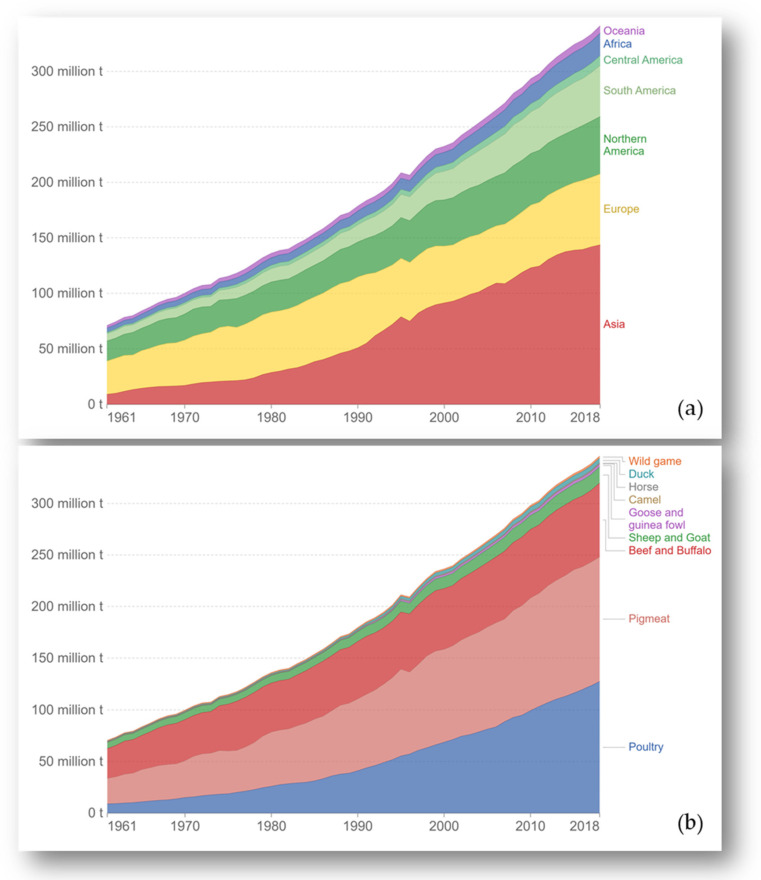
Global meat production (**a**) and meat production by livestock type (**b**). Source: https://ourworldindata.org/meat-production (accessed on 1 February 2022) [1].

Global meat production has increased rapidly over the past 50 years, with the rate of change across countries being highly variable [1]. Meat consumption growth has been most marked in countries that experienced a strong economic transition, for example, China and Brazil have grown approximately 15-fold and 4-fold, respectively, since 1961 (Figure 1). 

While worldwide in the 1960s, dietary protein primarily came from plant-derived products such as wheat, nowadays, up to 58% of the protein availability comes from animal-derived products. Consequently, at present, meat products constitute the major source of proteins in developed countries (28 g of protein/person/day), accounting for 30% of total energy consumption [2].

Among the different meat types available in the markets, poultry and pig meats have shown the highest increase in consumption [3,4] (Figure 1). Beef and buffalo meat consumption has remained stable in recent years, even decreasing slightly [4]. According to Salter et al. [5], in the period 2014–2016 total meat consumption per capita worldwide was 34.1 kg/year, being almost 60% red meats (pork, sheep and beef).

Nowadays, approximately 5% of the global population considers themselves vegetarian, whereas there are many more people (between 14 and 60%) that define themselves as flexitarian, which means that they reduce meat consumption, but it is not eliminated from the diet [6].

Diet is a key risk-modifying factor for chronic diseases, and it must be used appropriately throughout the key life stages such as during pregnancy, the menopausal period and in the elderly. In general, reducing risk in early life might be beneficial in later life. Consumption of meat and meat products provides a supply of important nutrients including proteins, iron and vitamins, among others—to the human body. As an essential part of a mixed diet, meat ensures adequate delivery of essential micronutrients and amino acids which are involved in the regulatory processes of energy metabolism for human health and development. However, despite being a key and unique combined source of high-quality proteins, iron and zinc, red and particularly processed meat has been associated with an increased risk of chronic diseases and pathological conditions.

Meat is defined as the flesh of an animal consumed as food. Red meat is considered all types of mammalian muscle (beef, veal, pork and lamb) which can be fresh, minced and frozen. Processed meat is defined as meat preserved by methods other than freezing, such as salting smoking, heating and marinating or by the addition of chemical preservatives (e.g., ham, bacon, hamburgers, salami, etc.) [7]. During the processing of meat, additional meat and animal fat may be added together with a wide range of non-meat substances and additives leading to more complex products. While the differences between meat types (red, white, different cuts, processed meats, etc.) may not be clear to many consumers, differential benefits and risks of chronic disease development and during key life processes between them are substantial.

This paper aims to summarize the benefits and challenges primarily associated with red and processed meat consumption including the risk of iron deficiency and chronic non-communicable diseases although some comparisons with white meat are included. In addition, the role of meat in the diet during pregnancy is examined as this period has major implications for the health of both mother and the unborn. 

## 2. Meat Consumption and the Risk of Iron Deficiency and Chronic Non-Communicable Diseases

### 2.1. Iron-Deficiency Anaemia

The World Health Organisation (WHO) [8] defines anaemia as the condition of having a low number of red blood cells (<4.7 and <4.2 million cells/µL for men and non-pregnant women, respectively) or a low blood concentration of haemoglobin (<13.0 and 12.0 g/dL for men and non-pregnant women, respectively). Iron-deficiency anaemia (IDA) is usually regarded as the most common cause of anaemia although differential diagnosis is important as there are other causes and there may be concurrent nutrient deficiencies and other health comorbidities. IDA particularly affects adolescent girls, women of childbearing age, pregnant women and children.

Iron is an essential nutrient being a component of haemoglobin in red blood cells and of myoglobin which transports oxygen around the body and stores it in muscles and other tissues, respectively. Iron is also involved with a number of enzymes that are involved in energy metabolism, the metabolism of nucleotides, and the synthesis of proteins and other metabolites [9]. Infants are generally associated with a higher risk of IDA due to their rapid growth and often limited dietary sources of iron. Iron is involved in many central nervous system processes that can affect infant behaviour and mental functioning which can persist into adulthood, potentially leading to lower productivity in adults [10]. There is increasing evidence of a relationship between iron and vitamin D such that low vitamin D status can contribute to a reduction in iron status and consequently increase the risk of anaemia [11]. The exact mechanism(s) for this interaction is not fully known but indications include vitamin D affecting erythropoiesis due to its effect on the iron regulatory hormone hepcidin [12] and having a direct effect on erythroid precursors in the bone marrow [13]. Overall, it seems clear that vitamin D status should always be examined when investigating IDA. Although bone marrow has been regarded as the best tissue for estimating iron storage in the body, it is invasive and costly to extract, and serum ferritin concentration is now generally accepted to provide a satisfactory measure of iron status.

WHO [14] reports that some 30% of the world’s population suffers from anaemia, predominantly IDA, with the highest prevalence in preschool-age children (47.4%) and pregnant women (41.8%). A high prevalence exists in many low- and middle-income countries, with India recording 60% and 54% in children and women (aged 15–49 years), respectively [15], and is a major concern. Poor iron status is however also seen in developed countries. In the United Kingdom, Year 8 of the rolling National Diet and Nutrition Survey [16] showed that about 25% of females in the age range 11–18 years had serum ferritin concentrations less than 15 μg/L with 12% of older females (19–64 years) also being of the sub-optimal status. Within that age range, pre-menopausal women will generally be of lower status than those that are postmenopausal. These indications of poor iron status in the UK reflect dietary iron intakes in the same study [16] with 54% of females 11–18 years and 27% of females 19–64 years having dietary iron intakes below the lower reference nutrient intake (4.1 mg/d; regarded as adequate for only the bottom 2.5% of the population).

Although the majority of worldwide anaemia is IDA, there is good evidence that other factors can contribute including intestinal parasite infestation as reported in India and in North American Inuits [17] where anaemia is a serious problem despite having a high meat intake.

Dietary iron can be in the form of haem or non-haem. Haem iron is only found in animal and seafood products, notably red meat and is highly bioavailable (typically 20–30% absorption) whereas non-haem iron is found in plant and animal-based foods and is much less bioavailable (typically 1–10% absorption). In addition, haem iron can increase the absorption of non-haem iron when they are consumed together in a meal [18] and this increase occurs even with diets rich in phytates that can inhibit iron uptake. Kristensen et al. [19] showed that including 60 g of Danish pork meat (classified as red) in high phytic acid diets (1250 µmol/d) increased the fractional absorption of non-haem iron compared with the same diets without the meat. The exact mechanism by which haem iron increases the absorption of non-haem iron is not entirely clear but appears to involve peptides and amino acids released from hydrolysis of the meat protein [20]. Anderson and Frazer [21] proposed that haem iron may bind to the enterocyte brush border and is then endocytosed into the enterocyte. Iron is then enzymatically released from haem and exported from the cells via ferroportin 1. Although the uptake of non-haem iron by the enterocyte is by a different process to haem iron, its export into the circulation is also via ferroportin 1 [21]. All this evidence highlights that those who consume red meat will get more highly available haem iron and some non-haem iron whereas vegetarians and vegans generally only get non-haem iron and thus have a higher risk of IDA. There is considerable variation in the iron concentration within and between meat types including fish (Table 1). Red meats and notably venison are generally the richest dietary source of iron with white meat and especially fish being considerably lower. 

**Figure 2:**
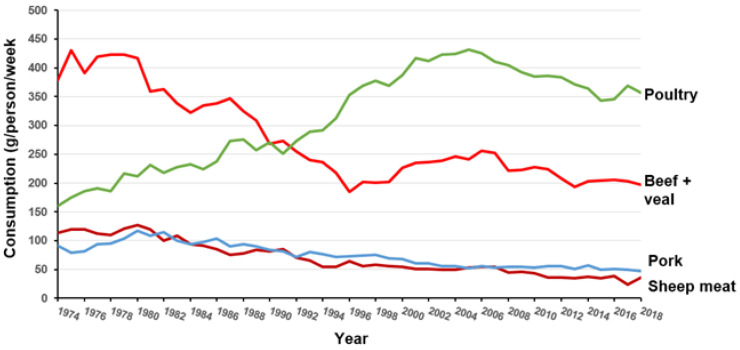
Changes in meat types consumed in the UK 1974–2018 (from DEFRA [25]).

Meat consumption in India has traditionally been low with the result that only about 1% of iron intake is in the form of haem [23]. There are large populations in India which are committed vegetarians. These groups have survived for possibly 2000 years following a “religious” prohibition of meat, probably developing adequate dietary strategies. The Indian National Family Health Survey 2015–2016 [15] reports that very few women consume meat daily although about 30% do consume it weekly. The considerably sub-optimal iron intakes in UK females noted above are primarily a reflection of a reduction in the consumption of red meat which has occurred over the last 40 years. Figure 2 shows the substantial reduction in consumption of red meat (beef, sheep meat and pork) and the rise in white meat (poultry) that has occurred in the UK from 1974 to 2018. Heath and Fairweather-Tait [24] reported that iron intake of UK adults fell from about 13.5 mg/person/day in 1970 to about 10 mg/person/day in 1998 which is similar to Years 7–8 of the rolling UK National Diet and Nutrition Survey [16] which reported mean intakes of 11.6 and 9.3 mg/d for men and women, respectively. Moreover, Roberts et al. [16] reported that for adults, meat now only supplies 21% of daily iron intake with the greatest source (38%) being cereals and cereal products emphasising the shift from haem iron to non-haem iron.

It is clear that red meat can be an important source of dietary iron but traditional dietary habits or reductions in habitual red meat consumption have led to sub-optimal iron intake with the attendant increase in the risk of IDA. The increased reliance on plant-based sources of dietary iron indicates the need for more attention to the concentration and bioavailability of this iron. It also suggests that maybe the use of red meat as an iron source should be targeted at children and young women.

### 2.2. Meat Consumption and Non-Communicable Chronic Diseases

Although mortality from cardiovascular diseases (CVD), including ischaemic heart disease (IHD) and stroke, is now declining in most of Europe, they remain the single largest cause of death worldwide, particularly in middle and later life. Moreover, there is considerable variability within Europe, for example in 2018, Bulgaria had 6.2 times the CVD mortality rate of France in 2016 [26]. CVD are also a substantial cause of morbidity and accounts for 82% of disability-adjusted life years [27]. In addition, since 1996, the number of people diagnosed with diabetes (predominantly type 2) in Europe has increased substantially and in 2019 Germany had the highest rate (15.3% of the population) with Ireland having the lowest at 4.4% of the population [28]. Other chronic diseases of concern are dementia and cancer. Due to the rapid ageing of many populations, the number of people living with dementia is projected to triple in the next 30 years [29].

Diet is a key risk-modifying factor for many chronic diseases and evidence for the association of meat consumption with various chronic diseases has been rather inconsistent. Indeed, the study of O’Connor et al. [30] provides evidence that heterogeneity in meat types hinders both the interpretation of meat intake and related chronic disease risk. This short review is focused on the chronic health effects of red meat, white meat and processed meat as defined by WHO [31] with most evidence based on data from long-term prospective studies.

#### 2.2.1. Cardiovascular Diseases

The meta-analysis of Micha et al. [32] reported that consumption of processed meat, but not red meat, was associated with a higher risk of IHD (Relative risk (RR) per 50 g/day: 1.42, 95% CI: 1.07, 1.89). The study of Bellavia et al. [33] with two Swedish cohorts reported that subjects in the highest quintile of red meat consumption had a 21% increased risk of all-cause mortality (hazard ratio (HR): 1.21, 95% CI: 1.13, 1.29) and a 29% increased risk of CVD mortality (HR: 1.29, 95% CI: 1.14, 1.46) compared with those in the lowest quintile. An early meta-analysis was carried out by Abete et al. [34] which involved 13 cohort studies that examined associations between total, processed, red and white meat intake and all-cause, CVD and IHD mortality. This found that those with the highest intake of processed meat had 22 and 18% higher risk of mortality from any cause and CVD, respectively, whilst red meat was associated with a 16% greater risk of CVD mortality. There were no associations for white meat. Looking at effects on hypertension, a major risk factor for CVD, particularly stroke, Schwingshackl et al. [35] undertook a systematic review and dose–response meta-analysis of prospective studies and reported a positive association between red meat intake and hypertension (RR 1.14 per 100 g/day, 95% CI, 1.02, 1.28). The risk from processed meat was about twice that for red meat (RR 1.12 per 50 g/day, 95% CI: 1.00, 1.26) although there were concerns about the quality of evidence from both meta-analyses.

These studies highlight the variability in outcome referred to above but more recently a number of systematic reviews and cohort studies with a large population have tried to provide more clarity. Iqbal et al. [36] reported on the association of unprocessed red and white meat and processed meat with mortality and CVD in the 21-country PURE study involving 134,297 participants with a 9.5-year follow-up. They found that high red meat intake (≥250 g/week vs. <50 g/week) was not significantly associated with total mortality or major CVD events and poultry meat intake was also not related to any of the assessed health outcomes. However, a high intake of processed meat (≥150 g/week vs. 0 g/week) was associated with a higher risk of total mortality (HR: 1.51, 95% CI: 1.08, 2.10, *p*-trend 0.009) and major CVD events (HR: 1.46, 95% CI: 1.08, 1.98, *p*-trend 0.004).

Due to the increasing number of systematic reviews, an additional form of evidence synthesis is the overview of systematic reviews (umbrella review). Jakobsen et al. [37] accordingly summarised three systematic reviews of cohort studies published 2017–2019 which included meta-analyses of associations between unprocessed red meat and CVD and stroke, unprocessed poultry meat and stroke and processed meat and CVD and stroke. A summary of the findings is given in Table 2. These indicate no clear associations between intakes of unprocessed red meat or processed meat and risk of CVD, an association between processed meat intake and higher risk of IHD and stroke, together with an association between poultry meat intake and a lower risk of stroke. However, using the NutriGrade system for grading meta-analysis evidence quality, only the processed meat intake and higher risk of IHD and stroke and unprocessed meat and stroke were graded as moderate quality, with the others being of low or very low quality. The authors concluded that many of the systematic reviews had critical weaknesses and future research should focus on CVD sub-types and substitution analysis between different meat types and between meat types and non-meat protein sources. Despite the weaknesses, a key outcome of the study is further evidence of the increased CVD risk linked with processed meat and possibly the neutrality of white meat.

#### 2.2.2. Type 2 Diabetes

The meta-analysis of Micha et al. [32] reported that processed meat intake was associated with a 19% higher risk of type 2 diabetes (RR: 1.19, 95% CI: 1.11, 1.27), although red meat consumption was not. The EPIC-InterAct study [38] with 340,234 adults in eight European countries found positive associations with type 2 diabetes linked to increased consumption of total meat (HR per 50 g/day: 1.08, 95% CI: 1.05, 1.12), red meat (HR per 50 g/d: 1.08, 95% CI: 1.03, 1.13) and processed meat (HR per 50 g/d: 1.12, 95% CI: 1.05, 1.19). However, these results cannot be extended to non-European countries in particular, and those with markedly different dietary histories and habits. Pan et al. [39] studied a male cohort of 26,357 and two female cohorts of 74,077 subjects with a 4-year follow-up and found that relative to a reference group with no change in red meat consumption, higher red meat intake by >0.5 portions per day was associated with a substantial rise in the risk of type 2 diabetes (HR: 1.48, 95% CI: 1.21, 1.41). When red meat intake was reduced by >0.5 portions per day a substantial reduction in risk was seen (HR: 0.86, 95% CI: 0.80, 0.93).

More recently Neuenschwander et al. [40] published an umbrella review of dose–response prospective cohort trials, involving unprocessed and processed red meat and processed meat generally, on the association with type 2 diabetes. They found no association for unprocessed red meat but substantially increased risk of type 2 diabetes from processed red meat (HR: 1.44, 95% CI: 1.18–1.76) and processed meat overall (HR: 1.37, 95% CI: 1.22–1.54).

Overall, the prospective study-based evidence on the association of red and processed meat intake with type 2 diabetes shows a positive association, particularly for processed meat although as with CVD, the findings are mixed, likely to be related to the variety of undefined meat types involved. Some supporting evidence was seen in a cross-sectional study with data from the UK National Diet and Nutrition Survey. Hobbs-Grimmer et al. [41] found a higher intake of processed red meat was significantly associated with higher glycated haemoglobin A1c (diagnostic marker for diabetes mellitus) concentration in women, whereas red meat showed no association. A similar effect was recorded by Ley et al. [42] who found higher haemoglobin A1c in females was associated with total, unprocessed and processed red meat consumption but suggested that this may have been related to a change in BMI which was not the case in the Hobbs-Grimmer et al. [41] findings. This review provides more evidence of a positive association of, in particular, processed meat with type 2 diabetes. Given the increasing prevalence of this condition its association with meat requires urgent attention.

The recent review on the effect of meat on human health by Geiker et al. [43] concluded that despite the availability of many prospective studies, a lack of clarity remains about the definition of meat types and uncertainty as to what degree residual confounding can explain the increased risk often associated with red and, particularly, processed meat consumption. Geiker et al. [43] recommended that ÿecognizeÿ controlled trials are needed to provide high-quality evidence on clearly defined meat intake. Although such studies rely on the use of markers of disease risk, they are clearly needed to resolve the current uncertain situation.

#### 2.2.3. Cancer

The World Cancer Research Fund (WCRF), the American Institute for Cancer Research (AICR) and the International Agency for Research on Cancer (IARC) are regarded as the key sources of authoritative evidence on the association between diet and cancer risk. They produce regular reports and updates as new evidence emerges. As a result, this section will mainly summarise their evidence for meat and cancer risk although other recent data will be cited.

WCRF/AICR [44] concluded in 2007 that the evidence was ‘convincing’ that red meat and processed meat were causes of colorectal cancer (CRC). The evidence was updated by WCRF/AICR [45,46,47] with additional data. The results from WCRF/AICR [46,47] are ÿecognizeÿ in Table 3 and broadly agree with the 2007 report. WCRF/AICR [47] concludes that there is ‘convincing evidence’ that processed meat increases the risk of colorectal cancer and ‘probable evidence’ that red meat does the same. Critically though, the data highlight that the risk of colorectal cancer associated with processed meat is approximately twice that of red meat. There were no other cancer types that had more than ‘limited suggestive’ evidence of having increased risk from meat consumption. A monograph from IARC [8] on the cancer risk from consumption of red and processed meat concluded that ‘there is sufficient evidence in humans for carcinogenicity of consumption of processed meat and causes cancer of the colorectum’. The report also concludes that ‘there is limited evidence in humans for carcinogenicity of consumption of red meat’ which is broadly similar to the WCRF/AICR [47] conclusion.

In response to the evidence of WCRF/AICR [45], the UK Government published public advice on meat consumption which remains today [48]. The advice for those who consume more than 90 g/d of cooked red and processed meat is to reduce this to 70 g/d. Perhaps concerning, the findings of Hobbs-Grimmer et al. [41] that 43% of UK adults (men 57% and women 31%) consume more than the 70 g/day guidelines. Given the differential evidence for red meat and processed meat, it would seem logical to have guidelines which ÿecognize this.

The recent study of Farvid et al. [49] is also of interest. This is a substantial systematic review and meta-analysis of prospective studies on cancer incidence relative to consumption of red meat and processed meat. They identified 148 papers that examined evidence on the association of red meat, processed meat and total red + processed meat and cancers at a wide range of sites. The RR data (highest vs. lowest) for colorectal cancers are shown in Table 3 and whilst broadly in line with the WCRF/AICR values, tend to show higher RR values for rectal cancers for all three meat groups. Farvid et al. [49] also reported significant positive associations for other cancers including lung (all three meat groups) and breast cancer (red meat, processed meat) which have not been highlighted by WCRF/AICR/IARC. The importance of these findings will need further consideration.

Overall, the findings provide further evidence on the association between red and processed meat and colorectal cancers with the evidence for processed meat being stronger than for red meat. However, it remains uncertain how the variability in the many meat types and their methods of processing around the world differ in the impact on cancer risk. It is also of note that the recent evaluation of Händel et al. [50] concluded that the recommendations to reduce intake of processed meat/meat products because of cancer risk were based on evidence which was not methodologically strong. In addition, the absolute health risk associated with meat consumption needs to be considered along with other lifestyle choices, perhaps especially with alcohol consumption which WCRF/AICR have shown to be substantially associated with cancers of the mouth, larynx and oesophagus. It remains very clear that considerably more research is needed in this area.

**Table 3:** Meta-analyses examining the relative risk (RR) and 95% confidence interval of colorectal cancers in relation to consumption of red and processed meat.

Meat Type	Colorectal Cancers RR per 100 g/day Red Meat/Red and Processed Meat and per 50 g/day Processed Meat ^1^			Colorectal Cancers RR Highest vs. Lowest Intake ^3^		
	All CRC	Colon	Rectal	All CRC	Colon	Rectal
Red and processed meat	1.10 (1.02–1.18)	1.19 (1.10–1.30)	1.17 (0.99–1.39)	1.17 (1.08–1.26)	1.21 (1.09–1.34)	1.26 (1.09–1.45)
Red meat	1.12 ^2^ (1.00–1.25)	1.22 (1.06–1.39)	1.13 (0.96–1.34)	1.10 (1.03–1.17)	1.17 (1.09–1.25)	1.22 (1.01–1.46)
Processed meat	1.16 ^2^ (1.08–1.26)	1.23 (1.11–1.35)	1.08 (1.00–1.18)	1.18 (1.13–1.24)	1.21 (1.13–1.29)	1.22 (1.09–1.36)

^1^ WCRF/ACIR [46] except where indicated; ^2^ WCRF/ACIR [47]; ^3^ Farvid et al. [49], RR values are highest vs. lowest.

#### 2.2.4. Dementia

The World Health Organisation (WHO) reports that more than 55 million people live with dementia across the world with some 10 million new cases each year [51] which is predicted to grow to 82 and 152 million by 2030 and 2050, respectively [52]. Alzheimer’s disease is the most common cause of dementia accounting for some 65% of cases. While an increasing age of many populations will account for some of the rises in cases, dementia is not a normal part of biological ageing with genotype, lifestyle factors and diet now known to contribute to dementia risk.

An earlier cross-sectional study [53] with data from Latin America, China and India claimed to be the first to show a positive association between meat consumption and dementia prevalence (prevalence ratio (PR) 1.19, 95% CI 1.07–1.31) although this was less consistent than the negative association between fish consumption and dementia (PR 0.81, 95% CI 0.72–0.91). This study did not have information on the type of meat consumed. Similar results associated with a transition to greater meat consumption have been seen in Japan with the strongest correlation involving a lag of 15–25 years [54].

The recent and perhaps most valuable evidence to date is based on a cohort study of some 490,000 subjects in the UK Biobank with a mean (± SD) follow-up period of 8 ± 1.1 years [55]. The key benefit of this study was the ability to examine the association with all-cause dementia risk according to the type of meat consumed. Each increment of 25 g/day of processed meat was associated with an increased risk of all-cause dementia (HR 1.44: 95% CI 1.24–1.67, *p*-trend < 0.011) and an increased risk of Alzheimer’s disease (HR: 1.52; 95% CI 1.18–1.96, *p*-trend = 0.001). Equivalent data per 50 g/day of red meat indicated decreased risks of all-cause dementia (HR: 0.81, 95% CI 0.69–0.95, *p*-trend = 0.011) and Alzheimer’s disease (HR: 0.70, 95% CI 0.53–0.92, *p*-trend = 0.009). The effect of unprocessed poultry meat was neutral with no association with the risk of all-cause dementia or Alzheimer’s disease. Similarly, there was no risk associated with total meat intake which clearly shows the importance of assessing the differential effects of the various meat types to avoid misinterpretation of the findings. This study also showed that subjects with the APOE ε4 allele had three to six times the risk of dementia although this did not significantly influence the associations with meat consumption. The study of Zhang et al. [55], although somewhat limited by a short follow-up period, highlights the increased dementia risk associated with processed meat, a product already linked with an increased risk of colorectal cancer. However as noted earlier, the components of processed meat can vary considerably, and research is needed to identify the compounds in processed meat that have causative functions for dementia. Nevertheless, this adds more evidence that processed and unprocessed meat should have separate considerations in public dietary guidance.

## 3. Meat Consumption and Key Life Processes

### 3.1. Skeletal Muscle and Bone Health and Maintenance

#### 3.1.1. Skeletal Muscle

Sarcopenia is a condition which can result from a chronic loss of muscle mass and muscle strength with advancing age [56]. A recent systematic review and meta-analysis [57] on the global prevalence of sarcopenia found it to vary between 10 and 27% in the studies used in the meta-analysis with the highest and lowest prevalence seen in Oceania and Europe, respectively. It is therefore a condition of primary relevance to the elderly although it is also seen in younger adults. The resulting loss of skeletal muscle mass can have major consequences because it is linked to reduced muscle strength and function. It also reduces the protection that muscle gives to bones leading to an increased risk of frailty and bone breakage often associated with falling. A less often recognised outcome of reduced muscle mass and a possibly associated reduced exercise ability is the increased risk of metabolic diseases, notably, type 2 diabetes. This is primarily a result of skeletal muscle insulin signalling being a major factor in glucose homeostasis [58].

It is now recognised that dietary protein and resistance exercise can provide an anabolic stimulus for skeletal muscle protein synthesis. There remain some doubts about the relative effects of the amount and type of protein although there is some consensus that protein intake by the elderly should be higher than given in many current recommendations [59]. There is also good evidence that the anabolic effect of protein can be enhanced by the consumption of proteins that have rapid digestion and absorption kinetics and that are also rich in leucine. As highlighted by Daly et al. [60], this has resulted in considerable research on the effects of milk proteins despite the evidence that red meat protein can also stimulate increased muscle protein synthesis at rest and after resistance exercise.

Daly et al. [60] reported a randomised controlled trial in 100 women aged 60 to 90 years which compared the effects of resistance exercise plus lean red meat (~160 g cooked, 6 days/week) with a control arm of resistance exercise plus 1 serving of rice or pasta/day on lean tissue mass, muscle size, strength and function. They concluded that a protein intake equivalent to about 1.3 g/kg body weight per day provided by lean red meat can give effective enhancement to the effects of resistance exercise on lean tissue mass and muscle strength. This was supported by the narrative review of Rondanelli et al. [61] which concluded that a balanced diet for sarcopenia prevention should include 113 g of meat (30 g protein) 4–5 times per week with the added recommendation that this should be made up of white meat two times/week, lean, red meat less than two times/week and processed meat less than once per week. The logic for including any processed meat in the guidelines is not clear.

Using data from the Framingham Offspring prospective study, Gorissen et al. [62] evaluated the effects of physical activity, high-protein foods and the combination of these over a nine-year follow-up on skeletal muscle mass and risk of functional decline. They concluded that men with higher intakes of animal-sourced proteins (red meat, poultry meat, fish and dairy) had higher skeletal muscle mass, regardless of exercise, than those who consumed plant sources. Moreover, of the less active participants, only those with reliance on animal-sourced proteins had a reduced risk of functional decline. The review [62] highlighted that despite a wide range of protein sources being consumed, in-depth data on the effects on muscle protein synthesis were only available for milk proteins, beef and the plant proteins in soya and wheat, with, to their knowledge, no studies on proteins in eggs, poultry, pork and fish. Their overall conclusions were that on a gram-for-gram basis, animal-derived proteins such as from milk and meat are more effective at stimulating muscle protein synthesis than plant proteins albeit with a higher environmental cost from beef in particular.

More recently Valenzuela et al. [63] reported on a systematic review and meta-analysis of randomised controlled trials comparing the responses of exercise training in combination with beef protein, whey protein or no protein on body composition and exercise performance. Seven studies were included in the meta-analysis although only four were suitable for comparing changes in body composition from beef and whey proteins and three studies for effects on muscle thickness. Overall, the study showed no mid/long-term (≥4 weeks) differences between beef protein and whey protein on protein intake, lean body mass and fat mass. A further conclusion was that since beef protein led to a significantly greater daily protein intake than no protein, beef protein may be an effective way of increasing lean body mass and lower limb muscle strength. It is unclear why the effect of whey protein intake was not significantly higher than no protein.

The study does highlight the continued lack of detailed comparisons, to the fractional muscle protein synthesis level, between beef and whey proteins and the lack of data relating to other meat types.

The studies to date are broadly indicative that beef protein can be as effective as whey protein for reducing the loss of skeletal muscle in the elderly. However, there remains a continued lack of detailed comparisons, to the fractional muscle protein synthesis level, between beef and whey proteins and indeed the lack of data relating to other meat types. Given the beneficial or neutral effects of white meat compared to the increased cancer risk associated with red and particularly processed meat, more work on the effects of white meat for maintaining skeletal muscle mass and strength in the elderly is certainly warranted.

#### 3.1.2. Bone Mass

The maintenance of bone mass is of great importance in the elderly and in postmenopausal women. In these categories, the risk of osteoporosis represents a major concern. Osteoporosis is a progressive systemic bone disease characterised by deterioration of bone tissue and micro-architecture leading to increased bone fragility and fracture [64]. Nowadays the increasing age of the global population may lead to a higher prevalence of osteoporosis and the incidence of osteoporosis-related fractures.

Meat foods besides being characterised by high protein, are also rich in phosphorus and magnesium. Prospective studies showed that individuals with higher protein intake have the slowest rate of bone loss. An increase in dietary protein is also known to increase circulating levels of insulin-like growth factor 1 which is a key mediator of bone growth. Magnesium influences mineral metabolism indirectly through a role in ATP metabolism and as a cofactor for more than 300 proteins, the calciotropic hormones and 1,25(OH)2D. Magnesium also influences bone health with direct effects on bone quality. Protein makes up roughly 50% of the volume of bone and about one-third of its mass, and this bone protein matrix undergoes continuous turnover and remodelling. Therefore, the regular consumption of proteins in the diet is a major factor influencing bone health in older individuals. However, a considerable amount of controversy concerning the relationship between dietary protein and bone metabolism remains. In particular, both high protein consumption without supporting calcium intake, and low protein intake (e.g., vegan diets) seem to be detrimental to the health of bone.

A study conducted on Chinese postmenopausal women demonstrated that high meat consumption was associated with improvements in skeletal metabolism in older individuals. The prevalence of OP was less frequent in women preferring meat food habits. In the study performed by Darling et al. [65], an increased protein intake (0.8–1.3 g/kg/day) demonstrated a benefit for bone health in healthy adults with no indication of any detrimental effect. Controversially, the effect of a high dietary ratio of animal to vegetable protein on bone health has been investigated in a cohort of 1035 women (Study of Osteoporotic Fractures, SOF) [66]. Women with a higher ratio of animal to vegetable protein intake had a higher rate of bone loss at the femoral neck than did those with a low ratio, as well as a greater risk of hip fracture. This suggests that a reduction in animal protein and an increase in vegetable protein may decrease bone loss and the risk of hip fracture although more evidence is clearly needed.

Red meat can provide an important contribution to micronutrient intakes among young infants, particularly around the time of weaning. At the time of weaning stores of some nutrients, such as iron, begin to be depleted indicating that additional dietary sources are needed. Red meat provides an important source of highly bioavailable iron. Therefore, young children should receive a variety of foods including meat, in particular when breastfeeding is continued instead of switching to formula or follow-on milk which will be fortified with iron [67].

Overall, meat protein is an important nutrient for bone health, although further research is required to clarify the quantities, food sources, timing and target population through which it exerts its positive influence.

#### 3.1.3. Meat Consumption during Pregnancy

The diet consumed during pregnancy may have implications for the health of both mother and the unborn infant. The nutritional environment that mothers provide during pregnancy is important for the optimal health, development, and long-term chronic disease risk of the infant [68]. General recommendations invite pregnant women to consume a balanced and varied diet consisting of frequent intakes of vegetables, fruit, whole grains, low-fat dairy, lean meat and fish, and legumes and nuts and limit the consumption of red and processed meat [69]. Anaemia during pregnancy, in particular in the third trimester, can cause adverse perinatal outcomes including preterm labour, premature rupture of membranes, and increased maternal and foetal mortality. Iron intakes among non-pregnant women of childbearing age are frequently below recommended levels with [16] reporting that in the UK 54% and 27% of females aged 11–18 years and 19–64 years, respectively, had iron intakes below the lower reference nutrient intake. This is likely to be the result of a chronic reduction in red meat consumption since as noted earlier, red meat is an excellent source of highly bioavailable haem iron.

Maternal diets before and during pregnancy could influence rates of preterm birth and infants born small for their gestational age (SGA). The latter have an increased risk of death and developmental and behavioural problems in childhood. Therefore, the importance of adequate maternal nutrition to reduce the risk of giving birth to SGA infants is of paramount importance in particular in developing countries.

Several reports describe that the dietary patterns characterised by higher intakes of processed meat were associated with a higher risk of having preterm infants. These diets contained pro-inflammatory nutrients, which act as a stressor on the hypothalamic-pituitary-adrenal system and might also be transferred through the placenta. Haugen et al. and Saunders et al. [70,71] reported that there was no association between Mediterranean diet consumption (characterised by intake of vegetables, legumes, fruits and nuts, cereals, fish, red meat and poultry, dairy products, alcohol and fat) and the risk of preterm birth. The consumption of processed red meat has been associated with an increased risk of gestational diabetes in infants [72]. The SUN project’s outcomes suggested that higher pre-pregnancy consumption of meat, especially red and processed meat, and haem iron intake, are significantly associated with increased gestational diabetes risk in pregnant women [73]. These outcomes were also confirmed by the study of Liang et al. [74] which indicated that higher dietary intakes of protein of animal origin in mid-pregnancy were associated with an increased risk of gestational diabetes among Chinese women. Cured meat is also recognised as the most important source of human N-nitroso compounds (NOC) exposure, due to high concentrations of nitrite that form around particles of cured meat in the stomach.

NOC formed in the stomach, between nitrites and secondary or tertiary amines or amides may be a major contributor to human cancer risk. In particular, transplacental exposure of one group of NOC, the nitrosoureas, causes neurogenic tumours in infants [75].

Furthermore, the gut microbiome has an important role in infant health and immune development and may be affected by early-life exposures. Maternal diet may influence the infant gut microbiome through vertical transfer of maternal microbes to infants during vaginal delivery and breastfeeding. The associations of red and processed meat with specific infant gut microbes showed that operational taxonomic units (OTUs) in the genus Bifidobacterium, generally recognised as a beneficial microbe, were decreased with increasing maternal fruit consumption in vaginally born infants yet increased with higher maternal red and processed meat consumption [76].

Overall, inconsistent findings have been observed in the association between maternal meat consumption and offspring health effects. The discrepancy of these findings might be due to the variety of measurement techniques, sample size and mother conditions.

## 4. General Conclusions

Red meat has been an important part of the human diet throughout human evolution. When included as part of a varied diet, it provides a rich source of high biological value proteins and essential nutrients, some of which (e.g., iron) are more bioavailable than in other food sources. It is suggested that the use of red meat as a protein and iron source should be targeted at children, young women and the elderly, especially in parts of the world where anaemia and childhood stunting are highly prevalent. There is, however, increasing evidence that processed meat is associated with increased risks of CVD, cancer and dementia. Red meat has a varied association with chronic disease risk and this area needs more clarity. White meat is neutral or associated with a lower risk of chronic diseases. Accordingly, there now seems little doubt that processed and unprocessed meat should have individual public dietary guidance linked to chronic disease risk. However, the implementation of this will need to further public guidance on different types of meat, ideally supported by additional studies to clarify the current uncertainties.

## Figures and Tables

**Table 1:** Typical iron concentrations in a range of meat types (from [22]).

Meat Type	Iron Concentration (mg/100 g)
Red meat	
Pork steaks, grilled, lean	1.1
Lamb leg steaks, grilled, lean	2.2
Beef rump steak, grilled, lean	3.6
Venison, roasted	5.1
White meat	
Turkey, light meat, roasted	0.50
Turkey, dark meat, roasted	1.2
Chicken, light meat, roasted	0.40
Chicken, dark meat, roasted	0.80
Fish	
Cod, flesh only, grilled	0.10
Haddock, flesh only, grilled	0.17
Salmon, flesh only, grilled	0.45

**Table 2:** Meta-analyses of associations between meat type and risk of CVD, IHD and stroke (from [37]).

Systematic Review Used	Number of Cohort Studies	Outcome	Comparison Used	Risk Ratio (95% CI ^1^)
Unprocessed red meat				
Zeraatkar et al. (2019)	3	CVD	Dose–response, per 50 g/day	1.01 (0.99, 1.02)
Kim et al. (2017)	6	Stroke	High vs. low intake	1.11 (1.03, 1.20)
Zeraatkar et al. (2019)	6	Stroke	Dose–response, per 50 g/day	1.01 (1.00, 1.01)
Unprocessed poultry meat				
Kim et al. (2017)	3	Stroke	High vs. low intake	0.87 (0.78, 0.96)
Processed meat				
Zeraatkar et al. (2019)	3	CVD	Dose–response, per 50g/day	1.01 (0.97, 1.05)
Bechthold et al., (2019)	3	IHD	Dose–response, per 50g/day	1.27 (1.09, 1.49)
Kim et al. (2017)	6	Stroke	High vs. low intake	1.17 (1.08, 1.25)
Bechthold et al. (2019)	6	Stroke	Dose–response, per 50g/day	1.17 (1.02, 1.34)
Zeraatkar et al. (2019)	6	Stroke	Dose–response, per 50g/day	1.02 (1.01. 1.04)

^1^ CI, Confidence interval.
